# Effects of mental fatigue on isometric mid-thigh pull performance and muscle activities

**DOI:** 10.1371/journal.pone.0318238

**Published:** 2025-03-12

**Authors:** Hyung Suk Yang, Lee T. Atkins, C. Roger James

**Affiliations:** 1 Division of Kinesiology and Sport Management, University of South Dakota, Vermillion, South Dakota, United States of America; 2 Center for Rehabilitation Research, Texas Tech University Health Sciences Center, Lubbock, Texas, United States of America; Portugal Football School, Portuguese Football Federation, PORTUGAL

## Abstract

This study investigated the effects of mental fatigue on rate of force development (RFD) and peak force during an isometric mid-thigh pull (IMTP), as well as its impact on muscle activation measured by electromyography (EMG) median frequency. Sixteen healthy, resistance-trained males completed two sessions: a control condition and a mentally fatigued state induced by a 30-minute modified Stroop task. IMTP performance and muscle activation were assessed before and after the mental fatigue task. Mental fatigue significantly reduced RFD in the later phase of force generation, specifically within the 20%-80% of maximum force interval (RFD_2080_) (*p* =  0.022, *d* =  0.638). In contrast, no significant changes were observed in RFD within the initial 0-100 milliseconds (RFD_100_) or 0-200 milliseconds (RFD_200_) of contraction, nor in peak force. Additionally, mental fatigue led to a significant increase in EMG median frequency for the rectus femoris during the initial 0-1 second interval (*p* =  0.040, *d* =  -0.609), with no significant changes in the medial gastrocnemius or other time intervals. These findings suggest that mental fatigue primarily impacts the later stages of force development, affecting the ability to sustain and develop force over time without compromising peak force. The increase in EMG median frequency for the rectus femoris indicates a possible compensatory response to mental fatigue, underscoring the complex influence of cognitive stress on neuromuscular function. This study highlights the importance of considering mental fatigue in activities requiring sustained or progressively increasing force production.

## Introduction

Mental fatigue is a psychophysiological state that occurs after or during prolonged periods of cognitive activity [1]. It is characterized by self-reported feelings of tiredness, lack of motivation, and decreased cognitive performance [2,3]. Mental fatigue is known to influence physical performance, particularly in tasks requiring sustained effort, by altering perceived exertion and neuromuscular control [4]. While previous research has examined the effects of mental fatigue on endurance tasks and maximal force production, its impact on isometric multi-joint strength tests, such as the isometric mid-thigh pull (IMTP), remains unclear. The IMTP is a widely used assessment for maximal lower-body strength and power, allowing for the measurement of critical performance metrics such as rate of force development (RFD), peak force, and muscle activation patterns. Investigating this relationship could provide insights into the interplay between mental and physical fatigue, with practical implications for optimizing performance and recovery strategies in athletic and clinical settings.

Several studies have examined the effect of mental fatigue on exercise performance and the perception of fatigue. Mental fatigue has been linked to decreases in endurance time, loss of peak power during cycling, and reductions in running velocity, running distance, and accuracy in tasks such as soccer shooting [4]. Additionally, mental fatigue has been found to reduce time to exhaustion and intermittent maximal force production over time [5,6]. Despite this, physiological markers such as heart rate, blood lactate, oxygen consumption, and cardiac output generally remain unchanged under mental fatigue [4,7]. Additionally, athletes frequently report higher perceived exertion when mentally fatigued, which can diminish performance even when physiological indicators do not reflect increased physical strain [4,8]. Similar findings have been documented in treadmill tests designed to simulate various sports activities, where mental fatigue negatively affected performance due to higher perceived exertion rather than physiological changes [9].

Conversely, during high-intensity, short-duration activities such as maximal isometric knee extensions, countermovement jumps, and sprints, individuals exhibit minimal performance decline under mental fatigue [7,9]. This suggests that mental fatigue primarily affects submaximal endurance tasks, where maintaining consistent effort and managing perceived exertion are crucial. Although some studies have found that mental fatigue does not impact muscle contractile properties or maximum force generation [6,10,11], other research presents conflicting findings with isometric single joint activities, suggesting potential impairments in neuromuscular function [12,13]. Specifically, mental fatigue might affect central factors such as motor cortex activity rather than just peripheral muscle properties [4,13]. This suggests that mental fatigue could influence the neuromuscular system in complex ways, necessitating further research to fully understand these mechanisms.

The IMTP is a widely used test to assess maximal strength and power in the lower body, providing valuable insights into an individual’s muscular capabilities and overall strength [14]. However, the interaction between mental fatigue and physical performance during complex, multi-joint isometric efforts like the IMTP has not been thoroughly investigated. Therefore, the purpose of this study was to investigate the effects of mental fatigue on IMTP performance and muscle activities. By exploring these interactions, this study aimed to contribute to a broader understanding of how mental fatigue influences physical performance and to inform practical approaches for mitigating its adverse effects. It was hypothesized that mental fatigue would impair IMTP performance by affecting neuromuscular function, altering motor control strategies, or reducing the capacity to maintain force output.

## Methods

### Participants

Sixteen healthy male participants aged 18-30 years were recruited for this study (age: 20.7 ±  1.4 years, height: 1.80 ±  0.08 m, mass: 83.6 ±  9.2 kg, BMI: 25.8 ±  2.7 kg/m^2^). The participants had been resistance-trained for a minimum of six months, engaging in resistance training 2-3 times per week. Potential participants were excluded from the study based on obesity (BMI over 30 kg/m^2^), any major musculoskeletal injury that could interfere with the IMTP, and any neurological impairment. Two participants were excluded from the electromyography (EMG) analysis due to technical errors. The study was approved by the Institutional Review Board of the University of South Dakota (IRB-22-54; date of approval: April 21, 2022). All participants provided written informed consent prior to participation. Participants were recruited in the period from 04.21.22 to 04.16.24.

### Procedure

This study employed a repeated-measures design. Each participant met with the research team once and completed both experimental conditions: one under a control condition (C1) and the other in a mentally fatigued state (C2). Participants were asked to bring their own shorts, a t-shirt, and athletic shoes for the session.

Upon arrival, participants were familiarized with the study procedures and received instructions on the Stroop task and visual analog scale (VAS). For EMG measurements, the skin on the right lower limb was cleaned to ensure proper electrode adhesion and signal quality. Two surface EMG electrodes (Delsys Trigno, Boston, MA) were then applied to the rectus femoris and medial gastrocnemius muscles.

Participants were encouraged to prepare for testing by engaging in approximately 2 minutes of light physical activity of their choice, which included walking, jogging, or stretching dynamically on their own. No structured warm-up protocol was provided. Following this, participants completed several practice trials of the IMTP to familiarize themselves with the task and ensure proper positioning and technique [15]. To establish baseline measurements, mental fatigue ratings were first assessed using the VAS (VAS 1), followed by an initial set of three IMTP trials performed under the control condition (C1). After the baseline trials, participants completed a 30-minute Stroop task to induce mental fatigue. Immediately after completing the Stroop task, mental fatigue levels were reassessed using the VAS (VAS 2). Participants then performed a second set of three IMTP trials under the mentally fatigued condition (C2). A one-minute rest interval was provided between IMTP trials in both conditions. All experimental tasks and conditions were completed during the same session.

### Isometric Mid-Thigh Pull testing (IMTP)

Participants performed the IMTP on two in-ground force plates (AMTI, Watertown, MA) to measure peak force and RFD. Participants assumed the standardized IMTP position, with both feet shoulder-width apart and positioned centrally on the force plates. The sagittal plane knee angle was set to 140 degrees (mechanical convention) using a goniometer, and participants maintained an upright trunk position throughout the task, as recommended for reliability in IMTP data collection [16–18].

A fixed barbell, secured to the ground using adjustable straps, was set to a height corresponding to each participant’s mid-thigh level. Before initiating the maximal pull, participants were instructed to first “set” by applying steady tension to the straps for approximately 3 seconds to take up the slack. During this quiet standing phase, they were reminded to remain relaxed and avoid precontraction until the next command [17,19,20]. Following the quiet standing phase, participants were given the command “Go!” and instructed to pull on the bar as hard and as fast as possible for six seconds. The verbal instruction was, “Pull as hard and as fast as possible until told to stop.” Standard lifting straps were used to prevent grip loss during the pulls, ensuring that grip strength did not limit force output [21,22]. Each participant performed three maximal-effort trials, with a one-minute rest interval between trials to minimize fatigue effects.

### Stroop task

During the mental fatigue session, participants engaged in a 30-minute modified Stroop color-word task, which has been recognized as an effective method to induce mental fatigue by requiring response inhibition and sustained attention in athletes of various levels [3,23]. This task involved four words (red, blue, green, and yellow) presented randomly across five sheets of paper, each containing 45 words. Participants were instructed to verbally state the ink color of each word, except when the ink color was red. For instance, if the word “yellow” was printed in green ink, they were to say “green.” However, when the ink color was red, participants had to say the printed word itself rather than the color. For example, if the word “blue” was printed in red ink, the correct response would be “blue.” Researchers monitored the task closely, instructing participants to restart the task if they made an error. Participants were encouraged to complete as many words as possible within the 30-minute duration. After completing the Stroop task, participants’ mental fatigue levels were reassessed using the VAS (VAS 2).

### Visual Analog Scale (VAS)

Participants’ subjective levels of mental fatigue were measured using a 100-mm VAS, a tool recognized for its validity and reliability in assessing mental fatigue [24]. The scale had descriptive labels at each end: “none at all” on one side and “maximal” on the other, with a 100-mm line connecting the two. Participants were asked to indicate their current level of mental fatigue by placing a mark on the line. The researcher then measured the distance from the left end of the scale to the participant’s mark, recording the measurement in millimeters.

### Instrumentation and data analysis

**Fig 1:**
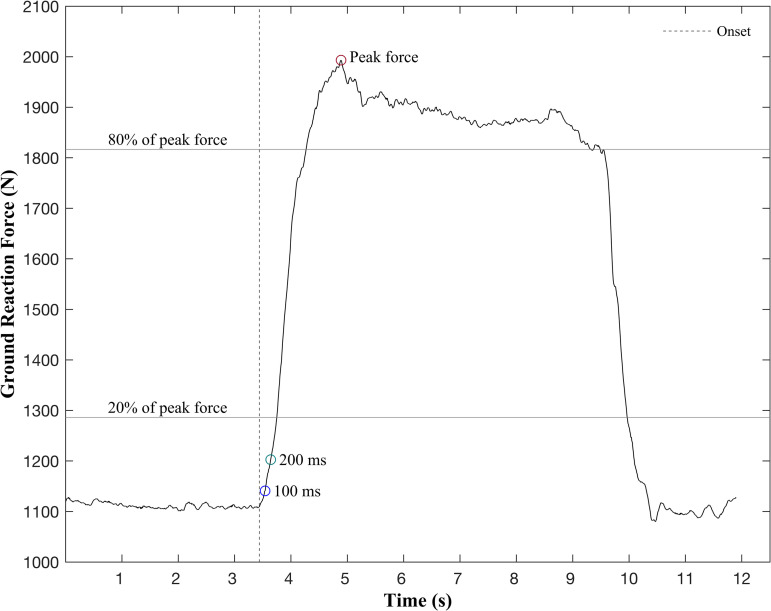
Illustration of ground reaction force parameters during isometric mid-thigh pull.

Force and EMG data were recorded using Vicon Nexus software at a sampling rate of 2000 Hz (Vicon, 2.13, Denver, CO). The data from Vicon Nexus were exported to MATLAB (Mathworks, Natick, MA) for further processing. Ground reaction force data were filtered using a fourth-order, zero-lag, Butterworth low-pass filter with a cutoff frequency of 50 Hz. Peak force was defined as the maximum force achieved during each IMTP trial (Fig 1). The RFD, which quantifies the ability to generate force rapidly, was calculated over specific time intervals. RFD_100_ represented the slope of the force-time curve during the first 100 milliseconds after contraction onset, while RFD_200_ represented the slope over the first 200 milliseconds. Additionally, RFD_2080_ was calculated as the slope of the force-time curve between 20% and 80% of the peak force achieved [25]. Contraction onset for each assessment was visually identified using force-time curves and the ginput function in MATLAB, a technique regarded as the criterion measure compared to automated methods [26]. For the EMG data, power spectral analysis was performed using custom MATLAB programs to assess muscle activation and fatigue. Median frequency, expressed in Hertz (Hz), was calculated for five consecutive one-second intervals starting at the onset of muscle contraction for the rectus femoris and medial gastrocnemius muscles. This parameter provides insight into muscle fatigue, as a decrease in median frequency over time can reflect changes in muscle firing patterns or recruitment strategies [27]. The EMG system featured an input impedance greater than 10 Gohms, a common mode rejection ratio exceeding 80 dB, and baseline noise less than 0.75 μV root mean square. The average of the three trials was used for the subsequent analysis of variables of interest.

### Statistical tests

Statistical analyses were conducted using Jamovi software (version 2.3.28.0, Sydney, Australia). Descriptive statistics (mean ±  standard deviation) were calculated for all variables. The normality of data distribution was assessed by examining Q-Q plots and performing Shapiro-Wilk tests. Paired sample t-tests were calculated to compare the variables between conditions. Effect sizes were calculated as Cohen’s *d* for paired samples, using the mean difference between conditions divided by the standard deviation of the differences between conditions [28,29]. Thresholds for interpreting the magnitude of effect sizes were small (*d* =  0.2), medium (*d* =  0.5), and large (*d* =  0.8). Additionally, 95% confidence intervals (CIs) were calculated for effect sizes to quantify the range within which the true effect size is likely to fall. Statistical significance was set at *p* <  0.05.

## Results

Normality of the data was confirmed for all variables using Q-Q plots and Shapiro-Wilk tests. There was a large increase in VAS scores from VAS 1 to VAS 2 (VAS 1: 19.5 ±  16.2 mm, VAS 2: 68.6 ±  20.5 mm, *d* [95% CIs] =  -2.56 [-3.58; -1.52], *p* <  0.001).

**Fig 2:**
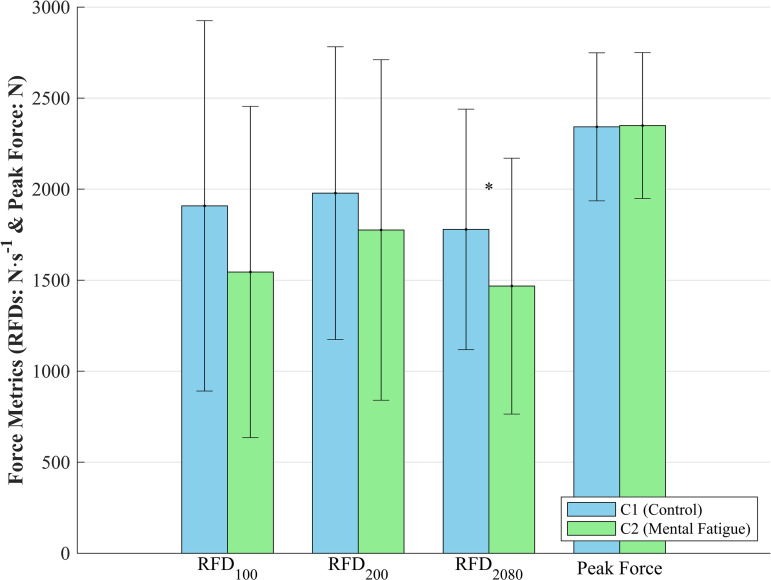
Force metrics (RFDs and peak force) for control and mental fatigue conditions. Mean ±  standard deviation of rate of force development (RFD) across three intervals (RFD100, RFD200, RFD2080) and peak force under control (C1) and mental fatigue (C2) conditions. Significant differences are indicated (*, p <  0.05). RFD is reported in N·s − ^1^ and peak force in N.

A medium-to-large decrease in RFD_2080_ was observed following the mental fatigue session (*d* [95% CIs] =  -0.64 [0.09; 1.17], *p* =  0.022; Table 1; Fig 2). However, no significant differences were found in RFD_100_ or RFD_200_ between conditions, nor were there significant changes in peak force between C1 and C2.

**Table 1:** Force metrics (mean ±  standard deviation) and statistical results.

Variables	C1	C2	Cohen’s *d*	95% CI Lower	95% CI Upper	*p* value
**RFD**_**100**_ **(N·s**^**-1**^)	1908.0 ± 1017.2	1544.8 ± 909.5	0.45	-0.07	0.96	0.091
**RFD**_**200**_ **(N·s**^**-1**^)	1978.3 ± 803.9	1775.4 ± 935.3	0.33	-0.18	0.83	0.210
**RFD**_**2080**_ **(N·s**^**-1**^)	1778.6 ± 661.0	1467.2 ± 702.6	0.64	0.09	1.17	0.022^*^
**Peak Force (N)**	2341.9 ± 406.5	2349.4 ± 400.7	-0.08	-0.57	0.41	0.749

Abbreviation: C1 =  Condition 1 (trials before the mental fatigue session), C2 =  Condition 2 (trials after the mental fatigue session), CI =  confidence interval, RFD_100_ =  rate of force development from 0 to100 milliseconds, RFD_200_ =  rate of force development from 0 to 200 milliseconds, RFD_2080_ =  rate of force development from 20% to 80% peak force time interval. A negative effect size indicates an increase in C2 compared to C1.

* : statistical significance (*p* <  0.05).

**Fig 3:**
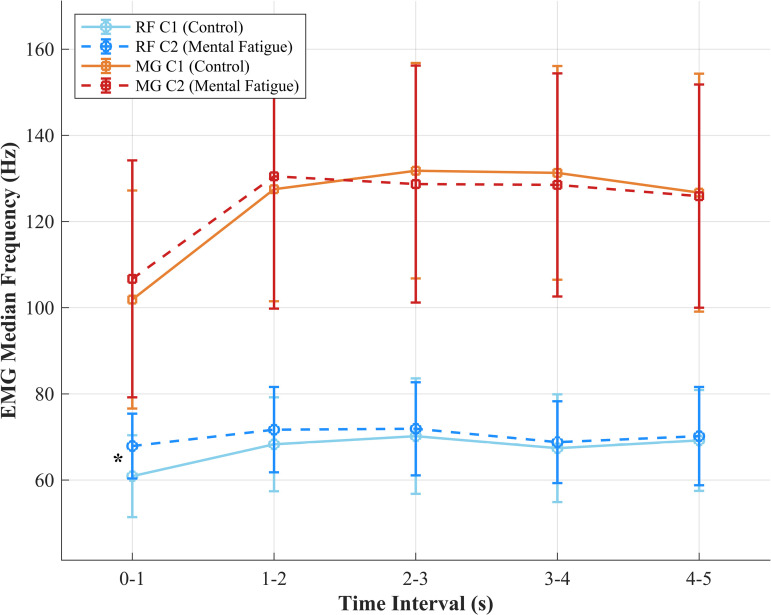
EMG median frequency for rectus femoris and medial gastrocnemius muscles across conditions. Mean ±  standard deviation of EMG median frequency for rectus femoris (RF) and medial gastrocnemius (MG) during five time intervals (0-1 s, 1-2 s, 2-3 s, 3-4 s, 4-5 s) under control (C1) and mental fatigue (C2) conditions. A significant difference is indicated (*, p <  0.05) for RF in the 0–1 s interval. EMG median frequency is reported in Hz.

For the rectus femoris, a moderate increase in median frequency was noted for the 0-1 s interval following the mental fatigue session (*d* [95% CIs] =  -0.61 [-1.17; -0.03], *p* =  0.040; Table 2; Fig 3). No significant differences were found in the other intervals. Similarly, for the medial gastrocnemius, there were no significant differences between C1 and C2 across any of the intervals.

**Table 2:** Median EMG frequency values (mean ±  standard deviation) and statistical results.

Variables	C1	C2	Cohen’s *d*	95% CI Lower	95% CI Upper	*p* value
**RF 0-1 s (Hz)**	60.9 ± 9.5	67.9 **± ** 7.5	-0.61	-1.17	-0.03	0.040^*^
**RF 1-2 s (Hz)**	68.3 **± ** 10.9	71.7 **± ** 9.9	-0.25	-0.78	0.29	0.370
**RF 2-3 s (Hz)**	70.2 **± ** 13.4	71.9 **± ** 10.8	-0.22	-0.75	0.31	0.419
**RF 3-4 s (Hz)**	67.4 **± ** 12.5	68.8 **± ** 9.5	-0.15	-0.68	0.38	0.582
**RF 4-5 s (Hz)**	69.2 **± ** 11.7	70.2 **± ** 11.4	-0.09	-0.61	0.44	0.749
**MG 0-1 s (Hz)**	101.9 **± ** 25.3	106.7 **± ** 27.5	-0.36	-0.89	0.19	0.205
**MG 1-2 s (Hz)**	127.5 **± ** 26.0	130.5 **± ** 30.7	-0.26	-0.79	0.28	0.344
**MG 2-3 s (Hz)**	131.8 **± ** 25.0	128.7 **± ** 27.5	0.30	-0.25	0.82	0.290
**MG 3-4 s (Hz)**	131.3 **± ** 24.8	128.5 **± ** 25.9	0.15	-0.38	0.68	0.576
**MG 4-5 s (Hz)**	126.7 **± ** 27.6	125.9 **± ** 25.9	0.04	-0.49	0.56	0.897

Abbreviation: C1 =  Condition 1 (trials before the mental fatigue session), C2 =  Condition 2 (trials after the mental fatigue session), CI =  confidence interval, RF =  rectus femoris, MG =  medial gastrocnemius. Data are presented for five sequential one-second intervals from the onset of contraction. A negative effect size indicates an increase in C2 compared to C1.

* : statistical significance (*p* <  0.05).

## Discussion

The purpose of this study was to investigate the effects of mental fatigue on the RFD and peak force during an IMTP, as well as its impact on muscle activation as measured by EMG median frequency. The results revealed that mental fatigue significantly reduced RFD during the later phase of force generation, specifically in the RFD_2080_ interval. However, mental fatigue did not significantly affect peak force, RFD_100_, or RFD_200_. Additionally, although the rectus femoris demonstrated a significant increase in median frequency in the initial phase (0-1 s) following the mental fatigue session, no significant changes were observed in the medial gastrocnemius or other EMG intervals.

The significant decrease in RFD_2080_ following the mental fatigue session suggests that mental fatigue primarily impacts the later phases of force development, where the ability to rapidly generate force becomes critical. RFD_2080_, which excludes the fluctuating portions at the beginning and end of the contraction phase (the first and last 20% of the contraction), enhances the reliability and precision of the measurement [25,30,31]. In our study, RFD_2080_ reflected the later stage of force development compared to RFD_100_ and RFD_200_, occurring before the first peak of the force. This finding aligns with previous research suggesting that mental fatigue can disrupt neuromuscular function and alter motor control strategies [32]. However, the lack of significant changes in RFD_100_ and RFD_200_ between conditions indicates that the initial, explosive phases of force production may be less susceptible to the effects of mental fatigue. This resistance to fatigue might stem from different neural mechanisms responsible for rapid force generation, which are possibly less affected by the cognitive demands imposed by mental fatigue. These findings suggest that while mental fatigue impairs the later stages of force development, it may not significantly hinder tasks that rely on short bursts of maximal effort.

Interestingly, mental fatigue did not significantly affect peak force in the IMTP. This suggests that maximal force generation may remain resilient to mental fatigue, likely due to the brief and intense nature of the effort involved. These findings align with previous research indicating that mental fatigue has minimal impact on high-intensity, short-duration activities [7,9]. This resilience could be attributed to physiological mechanisms that are less vulnerable to cognitive strain, or to participants’ ability to counteract fatigue through heightened effort or motivation during maximal exertion. The observed stability in peak force despite mental fatigue underscores the complexity of neuromuscular responses and suggests that while mental fatigue might impair sustained force production, it does not necessarily diminish maximal force output in more complex, multi-joint tasks like the IMTP. However, the conflicting findings in studies focusing on single-joint isometric activities [6,10,11] indicate that further research is needed to clarify the specific ways in which mental fatigue influences neuromuscular function across different types of physical tasks.

The significant increase in EMG median frequency in the rectus femoris during the initial 0-1 s interval of the IMTP in the mentally fatigued condition suggests that mental fatigue may induce changes in muscle activation patterns, possibly as a compensatory response. This increase could reflect a shift towards higher frequency motor unit recruitment or altered motor unit firing rates in reaction to the cognitive load imposed by the mental fatigue task. However, the lack of significant changes in the medial gastrocnemius and other time intervals implies that these effects might be muscle-specific or related to the timing of force application during the IMTP. Additionally, the absence of measurements from other key muscles involved in the IMTP, such as the hamstrings or gluteals, leaves open questions about whether similar changes in activation patterns occur across different muscle groups. These findings suggest the need for further research to explore how mental fatigue impacts a broader range of muscles, which could provide deeper insights into the overall neuromuscular adaptations to cognitive stress and their implications for physical performance.

The VAS results confirmed the presence of mental fatigue, with a significant increase in perceived fatigue levels recorded after completing the Stroop task. This increase in perceived fatigue aligns with the observed declines in RFD_2080_, reinforcing the connection between subjective feelings of fatigue and measurable decrements in neuromuscular performance.

One limitation of this study is that the order of conditions could not be randomized due to the lingering effects of the mentally fatiguing task. However, considering the straightforward nature of the IMTP and the participants’ familiarity with the exercise, along with the inclusion of practice trials, it is likely that the fixed order had minimal influence on the findings. Additionally, physical fatigue may have impacted performance, but with only three IMTP trials per condition and sufficient recovery time provided, the effect of physical fatigue is likely minimal. Finally, there is a possibility that participants expected the mentally fatiguing task to impair their force production (i.e., nocebo effect), which could have influenced the outcomes of the study.

These findings highlight how mental fatigue can affect physical performance, particularly by reducing the ability to quickly generate force during activities that require sustained or increasing effort over time. While peak strength appears to remain unaffected, the decreased ability to maintain rapid force production could have implications for tasks that demand consistent or prolonged effort, such as athletic performance or physically demanding work. The observed differences in muscle responses suggest that some muscles may adapt to mental fatigue differently, which could influence overall movement and coordination.

Understanding these effects can help athletes, coaches, and clinicians design training or recovery strategies to minimize the impact of mental fatigue. For example, tasks requiring rapid or sustained force production might benefit from mental recovery techniques or scheduling demanding activities during times of peak mental alertness. Further research is needed to explore how mental and physical fatigue interact and to identify strategies to mitigate their combined effects on performance.

## Supporting information

S1 Data This file contains individual participant data for perceived mental fatigue (VAS1 and VAS2), rate of force development (RFD) across intervals (RFD_100_, RFD_200_, RFD_2080_) and peak ground reaction forces (GRF_Peak) under control (C1) and mental fatigue (C2) conditions. It also includes EMG power spectral density (PSD) values for rectus femoris (RF) and medial gastrocnemius (MG) muscles, measured at five sequential 1-second intervals (0-1 s to 4-5 s) for both conditions. Columns provide descriptive and outcome variables for all participants.
(XLSX)
